# Supplementary material for: The interactome of intact mitochondria by cross-linking mass spectrometry provides evidence for coexisting respiratory supercomplexes
Source: Mol Cell Proteomics. 2017 Dec 8;17(2):216–32. doi: 10.1074/mcp.RA117.000470 (PMC5795388; doi:10.1074/mcp.RA117.000470)
Supplement: Supplemental Data [file supp_RA117.000470_133922_0_supp_23978_fzffwf.zip › spectra_annotation/mito_DR_spectra_annotation/489-1-3-1-3-1.pdf]

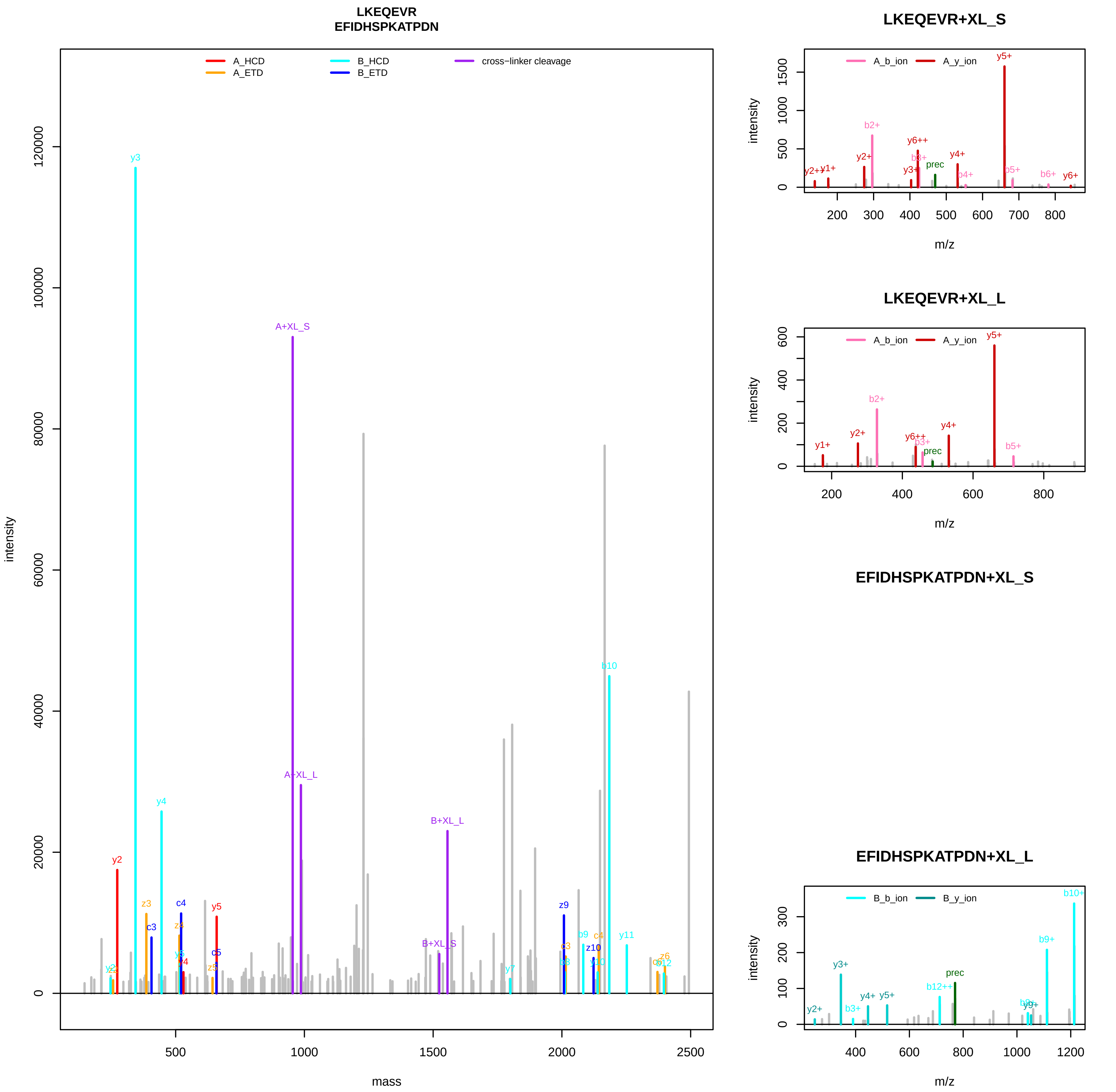
LKEQEVR
EFIDHSPKATPDN
A_HCD
A_ETD
B_HCD
B_ETD
cross−linker cleavage
intensity
mass
y3
A+XL_S
A+XL_L
B+XL_L
B+XL_S
y2
y4
z3
c3
c4
z4
y5
c5
z9
z10
b9
c4
y11
b10
c6
b12
z6
LKEQEVR+XL_S
A_b_ion
A_y_ion
y5+
b2+
y6++
y2+
y4+
prec
m/z
LKEQEVR+XL_L
EFIDHSPKATPDN+XL_S
EFIDHSPKATPDN+XL_L
B_b_ion
B_y_ion
b10+
b9+
y3+
b12++
y4+
y5+
